# N-Doped Reduced Graphene Oxide/Gold Nanoparticles Composite as an Improved Sensing Platform for Simultaneous Detection of Dopamine, Ascorbic Acid, and Uric Acid

**DOI:** 10.3390/s20164427

**Published:** 2020-08-07

**Authors:** Daria Minta, Zoraida González, Piotr Wiench, Stanisław Gryglewicz, Grażyna Gryglewicz

**Affiliations:** 1 Department of Process Engineering and Technology of Polymer and Carbon Materials, Faculty of Chemistry, Wrocław University of Science and Technology, Gdańska 7/9, 50-344 Wrocław, Poland; daria.minta@pwr.edu.pl (D.M.); piotr.wiench@pwr.edu.pl (P.W.); 2 Instituto de Ciencia y Tecnología del Carbono, INCAR-CSIC, Francisco Pintado Fe 26, 33011 Oviedo, Spain; zoraidag@incar.csic.es; 3 Department of Engineering and Technology of Chemical Processes, Faculty of Chemistry, Wrocław University of Science and Technology, Smoluchowskiego 25, 50-372 Wrocław, Poland; stanislaw.gryglewicz@pwr.edu.pl

**Keywords:** electrochemical sensor, simultaneous detection, dopamine, interference, nitrogen-doped reduced graphene oxide, gold nanoparticles

## Abstract

Gold nanoparticles (AuNPs) were homogeneously electrodeposited on nitrogen-doped reduced graphene oxide (N-rGO) to modify a glassy carbon electrode (GCE/N-rGO-Au) in order to improve the simultaneous detection of dopamine (DA), ascorbic acid (AA), and uric acid (UA). N-rGO was prepared by the hydrothermal treatment of graphene oxide (GO) and urea at 180 °C for 12 h. AuNPs were subsequently electrodeposited onto the surface of GCE/N-rGO using 1 mM HAuCl_4_ solution. The morphology and chemical composition of the synthesized materials were characterized by field-emission scanning electron microscopy and X-ray photoelectron spectroscopy. The electrochemical performance of the modified electrodes was investigated through cyclic voltammetry and differential pulse voltammetry measurements. Compared to GCE/rGO-Au, GCE/N-rGO-Au exhibited better electrochemical performance towards the simultaneous detection of the three analytes due to the more homogeneous distribution of the metallic nanoparticles as a result of more efficient anchoring on the N-doped areas of the graphene structure. The GCE/N-rGO-Au-based sensor operated in a wide linear range of DA (3–100 µM), AA (550–1500 µM), and UA (20–1000 µM) concentrations with a detection limit of 2.4, 58, and 8.7 µM, respectively, and exhibited satisfactory peak potential separation values of 0.34 V (AA-DA), 0.20 V, (DA-UA) and 0.54 V (AA-UA). Remarkably, GCE/N-rGO-Au showed a very low detection limit of 385 nM towards DA, not being susceptible to interference, and maintained 90% of its initial electrochemical signal after one month, indicating an excellent long-term stability.

## 1. Introduction

Dopamine (DA) is one of the major catecholamine neurotransmitters in the human body, showing a fundamental role in metabolism and controlling the central nervous, hormonal, and cardiovascular systems [1,2,3]. An inadequate level of DA in an organism leads to neurological disorders such as Parkinson’s and Alzheimer’s diseases or HIV infection [4,5]. As the level of DA can reflect health status [6], much effort has been put into development of fast, simple, and low-cost methods of DA detection. Even though electrochemical methods have shown great promise, DA usually coexists with ascorbic acid (AA) and uric acid (UA), which also play a significant role in human body [2,7,8]. AA is commonly used in common cold treatment [2] while UA, as a primary product of purine metabolism, may cause pneumonia and gout in unusual concentrations [2,7]. Both AA and UA exhibit similar oxidation potentials to that of DA, thus resulting in overlapped voltammetric responses [3]. This limitation can be mostly overcome by modifying the sensor with graphene materials, metal nanoparticles, and/or their composites [2,5,8]. It brings a great potential of electrochemical detection for the simultaneous and selective detection of DA, AA, and UA with high sensitivity, accuracy, reproducibility, and stability.

Graphene and graphene-related materials, such as reduced graphene oxide (rGO), have promising properties, such as good electrical and thermal conductivity, large surface area, high electrochemical activity, and ease of functionalization, that makes them suitable electrode materials in electrochemical detection of DA [9,10,11,12]. Regarding the synthesis of rGOs, various reduction processes have been proposed to restore the electrical properties by removing the oxygen groups of the initial graphene oxide (GO) [13,14]. Among them, hydrothermal treatment of GO is one of the strategies commonly followed, due to its low cost, simplicity, environmentally friendly quality, and efficiency at oxygen removal [15]. Additionally, this procedure does not rely on toxic and dangerous reducing agents such as hydrazine, which is commonly used [14]. Aiming to enhance the electrochemical sensing characteristics of these rGOs, several authors have proposed their modification with different metallic nanoparticles. Among them, gold nanoparticles (AuNPs) have attracted considerable attention because of their chemical stability [16,17] and well-developed electrochemically active surface area [18,19]. In addition, the electron transport properties of the as-obtained rGOs can be further improved by nitrogen doping [20,21]. We have previously demonstrated that the hydrothermal treatment of GO in the presence of N-dopant results not only in the introduction of nitrogen functional groups but also in a much higher degree of deoxygenation than that obtained in the absence of such N source [22]. Furthermore, the electrochemical performance of resulted N-rGOs towards DA detection was significantly improved as a consequence of the addition of electrons to the sp^2^ conjugated system [23,24,25]. 

Even though most recent research reports have been focused on the development of electrochemical sensors for selective detection of DA [5,8], there is an increasing interest in development of improved materials that allow the simultaneous detection of DA, AA, and UA. Thearle et al. [26] introduced nitrogen to the structure of rGO using plasma containing ammonia under microwave irradiation. The resulting N-rGOs performed well in the separate detection of DA, AA, and UA. However, simultaneous detection was confounded by overlapping DA and UA peaks. Wang et al. [2] used AuNPs to modify the surface of rGO. A GCE was electrochemically modified in two steps, consisting of the reduction of GO to rGO followed by the deposition of AuNPs from an aqueous solution of HAuCl_4_. The resulting electrode was able to simultaneously detect DA, AA, and UA, with a detection limits (LODs) of 1.4, 51, and 18 µM, respectively. Nevertheless, these detection limits were determined as the concentrations of all species increased at the same time. This approach does not provide a full picture of the impact of interferences on the detection of the main analyte. Tiğ et al. [3] proposed composite consists of AuNPs, GO and poly(2,6-pyridinedicarboxylic acid) (P(PDA)) in simultaneous detection of DA, AA, and UA. Firstly, they modified GCE electrochemically with AuNPs, and then they performed electrodeposition of P(PDA)-GO film on the surface of previously prepared GCE/AuNPs. In the electrochemical measurements, they obtained three peaks from DA, AA, and UA with peak potential separations of 0.161 (AA-DA), 0.336 (AA-UA), and 0.175 V (DA-UA). 

In this work, we investigated the performance of N-rGO-Au materials in the detection of DA and its coexisting analytes AA and UA in comparison with commonly used rGO-Au. To the best of our knowledge, this is the first study directly comparing both active materials towards the simultaneous detection of the mentioned analytes. The benefit of N-doping of rGO on the subsequent deposition of AuNPs was demonstrated. GCE/N-rGO-Au performed better than GCE/rGO-Au, which can be explained by the higher concentration and uniform distribution of AuNPs on the surface of the nitrogen-doped rGO, resulting in improved electrical conductivity and the protection of nitrogen groups against oxidation. The low detection limits obtained for AA and UA are in agreement with the suitability of N-rGO-Au for the simultaneous detection of DA, AA, and UA. Additionally, the proposed electrode (GCE/N-rGO-Au) is highly stable and exhibits superior selectivity in DA detection at high concentrations of AA and UA, which represents a significant step forward in development of the advanced electrochemical sensors.

## 2. Materials and Methods

### 2.1. Preparation and Characterization of Graphene-Based Materials

GO was obtained by a modified Hummers method previously described [27]. Briefly, 2 g of graphite (C-NERGY KS 6L, TimCal) was mixed in a flask with 96 mL of 98% H_2_SO_4_ and 2 g of NaNO_3_ (Sigma-Aldrich). Next the flask was cooled to 7–9 °C in an ice bath and 12 g of KMnO_4_ (Sigma-Aldrich) was added to the mixture under constant stirring. Then, the mixture was heated to 35 °C and stirred for 3 h. After that, 400 mL of H_2_O_2_ (3 wt.%, Sigma-Aldrich) was added. The resulting graphite oxide (GrO) was washed with Milli-Q water until the supernatant reached a neutral pH. Finally, GrO was exfoliated in a sonication bath for 2 h to obtain a GO aqueous suspension.

N-rGO was prepared by treating the previously obtained GO with urea (Sigma-Aldrich) under hydrothermal conditions. 100 mL of GO suspension (1 mg mL^−1^) was mixed with 1 g of urea and placed in an autoclave (PARR4848, Parr Instrument Company). The reaction was performed at 180 °C for 12 h with a constant stirring of 200 rpm [20]. The resulting product was washed with Milli-Q water and isopropanol and subsequently vacuum-dried overnight at 60 °C. For comparative purposes, non-doped rGO was produced by the same procedure in the absence of N-dopant.

Field-emission scanning electron microscopy (FESEM, FEI Quanta 650 FEG) was used to examine the morphology of graphene materials and the dispersion of AuNPs on their surfaces. The surface composition and distribution of nitrogen and oxygen functional groups of the graphene materials were determined by X-ray photoelectron spectroscopy (XPS). The C1s and N1s core-level spectra were deconvoluted using CasaXPS software into four and five separated peaks, respectively. The C1s spectrum was resolved into sp^2^ hybridized carbon (284.5 eV), hydroxyl, epoxy and C-N groups (286.5 eV), carbonyl and quinone bonds (287.6 eV), and carboxylic groups (289.0 eV). The components of the N1s core-level spectrum were resolved into pyridinic-N (N6, 398.7 eV), amide, amine and lactams (NC, 399.7 eV), pyrrolic-N (N5, 400.3 eV), quaternary-N (401.4 eV), and pyridine N-oxides (402–405 eV). 

### 2.2. Preparation of Nanocomposites by AuNPs Electrodeposition 

Four milligrams of N-rGO or rGO was mixed with 1 mL dimethyloformamide (DMF, Sigma-Aldrich) and Milli-Q water (*v*/*v* of 1:1) and ultrasonicated for 3 h to obtain a homogenous dispersion. GCE was polished with 0.3 and 0.05 µm alumina slurries and washed with water. The as cleaned GCE was then modified by drop casting of 2.5 µL of N-rGO or rGO dispersion and dried under an infrared lamp. Subsequently, the AuNPs were electrodeposited onto GCE/N-rGO or GCE/rGO in 1 mM HAuCl_4_ (Sigma-Aldrich) solution using cyclic voltammetry (CV) in a potential range of −0.1 and −0.9 V (vs. SCE) at a scan rate of 50 Mv·s^−1^. Two series of GCE/N-rGO and GCE/rGO-based modified electrodes were fabricated, changing the number of electrodeposition cycles from 10 to 40. Their electrochemical performance was evaluated based on the measurement of the anodic current in a 0.1 M PBS solution (pH 7.4) containing 100 µM of DA (Figure S1). The optimized number of electrodeposition cycles was applied (20 cycles for GCE/N-rGO and 30 cycles for GCE/rGO). The prepared electrodes were denoted as GCE/N-rGO-Au and GCE/rGO-Au, respectively.

### 2.3. Characterization of the Electrochemical Performance of the Sensors 

All electrochemical measurements were conducted in a three-electrode cell using a VMP3 potentiostat–galvanostat (BioLogic Science Instruments, France). The cell consisted of GCE/N-rGO-Au or GCE/rGO-Au, a platinum wire and a saturated calomel electrode (SCE) as working, counter, and reference electrodes, respectively. The electrochemical measurements were performed in a 0.1 M phosphate buffer solution as a supporting electrolyte. Cyclic voltammetry (CV) measurements were performed in a potential range of −0.5 to 0.8 V vs. SCE (i.e., 0.244 V vs. NHE) at a scan rate of 100 Mv·s^−1^. The calibration curves were obtained by differential pulse voltammetry (DPV) with optimized parameters. Pulse width, increment, and period were identical for both electrodes and were set to 25 ms, 5 mV, and 50 ms, respectively. The pulse heights for GCE/N-rGO-Au and GCE/rGO-Au were set to 100 and 125 mV, respectively.

## 3. Results and Discussion

### 3.1. Morphological and Structural Characterization of the Materials

**Figure 1:**
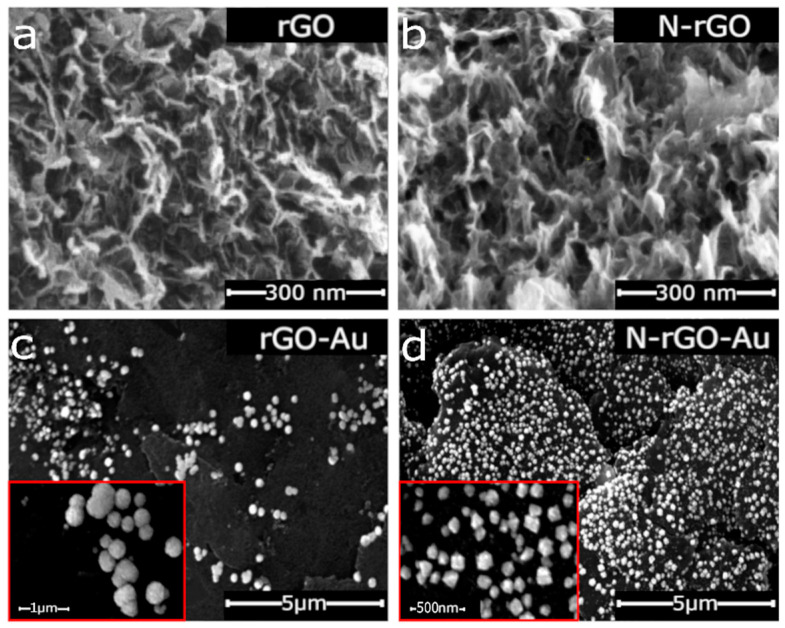
FESEM images of (**a**) rGO, (**b**) N-rGO, (**c**) rGO-Au, and (**d**), N-rGO-Au. Insets on (**c**,**d**) show enlarged areas of the corresponding materials containing AuNPs.

The FESEM images of the synthesized graphene materials (rGO, N-rGO) and their nanocomposites with AuNPs are shown in Figure 1. As expected, rGO and N-rGO (Figure 1a,b) exhibit a comparable flame-like morphology consisting of aggregated and wrinkled graphene sheets of comparable thickness [20,28]. However, there are remarkable differences after the electrodeposition of the metallic nanoparticles, depending on the graphene material selected as a support. As it can be seen in Figure 1d, a higher amount of these nanoparticles homogeneously distributed is present on the surface of N-rGO when comparing with r-GO (Figure 1c), despite a lower number of electrodeposition cycles (see Experimental section). Moreover, according to the insets of both images, AuNPs deposited on the N-rGO-Au surface consist of Au tetrahedral nanostructures, whereas AuNPs formed on the rGO surface are spherical. These results are in agreement with the previous reports, where the preferential deposition of AuNPs on the N-rich areas of graphene materials have been described [29,30,31]. The nitrogen functional groups in the graphene nanosheets are supposed to be responsible for the anchoring and reduction of gold cations. Moreover, the smaller size of the AuNPs deposited on the N-rGO surface (20–250 vs. 50–450 nm for rGO-Au) also suggests that the N functional groups prevent their aggregation.

The surface chemical composition of the synthesized materials as determined by XPS confirmed the higher AuNPs content in N-rGO-Au (7.6 vs. 6.2 at.% for rGO-Au (Table 1)). The initial nitrogen content in N-rGO (6.4 at.%) decreased to 2.7 at.% in N-rGO-Au, while the O content remains unaffected, thus indicating that nucleation and growth of AuNPs proceed preferentially on the nitrogenated groups.

Further information on the type and amount of functional groups of the synthesized materials (Table 2) was provided by deconvolution of the C1s and N1s core-level XPS spectra (Figures S2 and S3 in the Supplementary Materials).

As expected, among oxygen groups, C-O linkages (hydroxyl and epoxy) are the most abundant in all rGO-based samples. Notably, the intensity of the peak at a binding energy of 286.5 eV for N-doped graphene materials is higher than that of undoped rGO, which is due to the contribution of C-N bonds. The lower content of carboxylic groups in N-rGO compared to rGO can be explained by their improved removal during hydrothermal treatment of GO in the presence of N-dopants [22,32]. 

Nitrogen atoms were incorporated into the graphene structure in five configurations. Pyridinic nitrogen groups were the main functionalities in the structure of N-rGO (2.6 at.%), constituting 40.6 % of the total nitrogen content (Table 2), while a slightly lower amount (1.9 at.%) of pyrrolic N was also found. The other nitrogen functionalities in N-rGO in decreasing order are: quaternary N, amide/amine/lactam and oxidized pyridinic N. After Au electrodeposition, the amount of nitrogen functional groups on the N-rGO-Au surface decreased by more than twofold, which proves that AuNPs were preferentially deposited in the N-doped regions. Moreover, nitrogen in the form of pyridine N-oxide, which is present in the N-rGO sample (0.5 at.%), was not detected on the N-rGO-Au surface, suggesting that pyridine N-oxide is involved in anchoring the Au ions, presumably due to its complexation ability [33]. 

### 3.2. Electrochemical Sensing

#### 3.2.1. Preliminary Evaluation of the Electrochemical Performance of the Electrodes Towards DA, AA, and UA Detection

**Figure 2:**
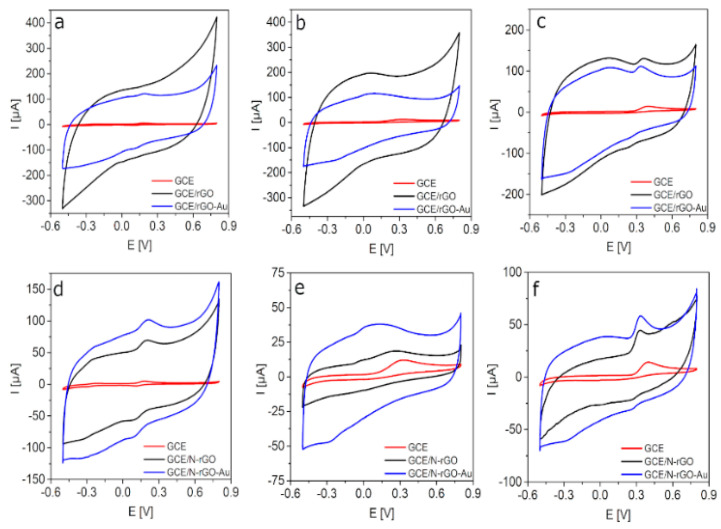
CVs recorded on (**a**–**c**) GCE, GCE/rGO, GCE/rGO-Au and (**d**–**f**) GCE, GCE/N-rGO, and GCE/N-rGO-Au electrodes in 0.1 M PBS (pH 7.4) containing (**a**,**d**) 100 µM of DA, (**b**,**e**) 300 µM of AA, and (**c**,**f**) 300 µM of UA.

As the first step, the electrochemical behavior of the different electrodes towards DA, AA, and UA sensing was evaluated by means of CV measurements. Figure 2 shows the CVs recorded on both GCE/rGO (Figure 2a–c) and GCE/N-rGO (Figure 2d–f) electrodes prior to and after the electrodeposition of the metallic nanoparticles (the bare GCE was also shown for comparison purposes) in 0.1 M PBS solution (pH 7.4) containing 100 µM DA (Figure 2a,d), 300 µM AA (Figure 2b,e) and 300 µM UA (Figure 2c,f). The bare GCE exhibits a negligible response to DA oxidation (5.2 µA at 178.2 mV), which is slightly improved after its modification with rGO-Au, because a small oxidation wave together with an important capacitive current are being developed. However, the electrochemical performance of the bare electrode was significantly enhanced after its modification with the N-doped materials [32], especially in the case of the composite with AuNPs showing a well-developed DA oxidation peak at 212.1 mV with an anodic Faradaic current of 101.9 µA. Regarding measured capacitive current, the corresponding values were lower for GCE/N-rGO and its composite with AuNPs that could represent an advantage when comparing with the GCE/rGO and GCE/rGO-Au electrodes also evaluated. Similar behaviors were observed in the preliminary evaluation of AA and UA on the different electrodes. The response to AA and UA oxidation recorded on the bare GCE was almost negligible (12.1 µA at 325.9 mV for AA and 14.2 µA at 401.9 mV for UA). After its modification with rGO-Au nanocomposite, only slight changes in oxidation potentials of AA and UA (115.9 µA at 105.1 mV and 111.6 µA at 336.8 mV) were observed. In the second step, the modification of the GCE with N-rGO nanocomposites allowed obtaining the well-developed AA and UA oxidation peaks at 274.8 mV (18.6 µA) and 334.7 mV (44.7 µA), respectively. However, the subsequent modification of the GCE with N-rGO-Au resulted in an improved electrochemical performance. The oxidation peak potential of AA was decreased to 80.4 mV and an improved anodic peak current of 38.1 µA was measured. Moreover, the anodic peak current of UA increased up to 58.4 µA (334.8 mV). 

#### 3.2.2. Optimization of the Operational pH

**Figure 3:**
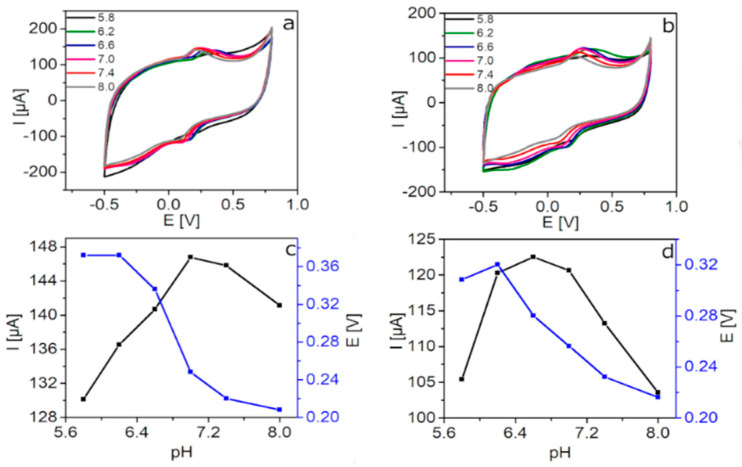
CVs recorded on (**a**) GCE/rGO-Au and (**b**) GCE/N-rGO-Au electrodes at different pH values in 0.1 M PBS containing 100 µM DA. Effect of the pH on the anodic peak current and anodic peak potential value of the oxidation of DA for (**c**) GCE/rGO-Au and (**d**) GCE/N-rGO-Au.

The optimization of the pH of PBS-based solution is crucial to achieve the better electrochemical response of the electrodes towards DA, AA, and UA sensing. In this regard, the influence of this parameter on the DA oxidation process was studied by means of CV measurements at pH values ranging from 5.8 to 8.0 (Figure 3). Both GCE/rGO-Au (Figure 3a) and GCE/N-rGO-Au (Figure 3b) present an optimized pH value of 7.0 (very close to the physiological value of 7.4 [34]). Better developed oxidation peak (120.7 µA at 256.4 mV) and lower capacitive current are observed for the N-doped material. Even though the maximum anodic current was measured on GCE/N-rGO-Au at pH 6.6 (Figure 3d), the above mentioned pH 7.0 was selected as the optimum one, looking for a compromise between the anodic current measured and the overpotential of the DA oxidation. Similar measurements were performed towards AA and UA oxidation (Figures S4a,b and S5a,b) on both modified electrodes. The values of the anodic peak currents corresponding to AA and UA oxidation recorded on GCE/rGO-Au were similar in the 5.8–7.0 pH range. However, in Figures S4c and S5c, it is clearly visible that lowest oxidation peak potentials were recorded at pH 7.0 (95.2 µA at 139.1 mV for AA and 78.9 µA at 380.9 mV). A comparable behavior was observed applying GCE/N-rGO-Au towards AA and UA detection (Figures S4d and S5d). The anodic peak currents corresponding to AA and UA oxidation recorded at pH 7.0 were 38.6 µA (70.8 mV) and 35.5 µA (326.8 mV), respectively. As mentioned before to find a compromise between the anodic peak current and the overpotential considering the oxidation of DA, AA, and UA, the pH 7.0 was selected as a suitable value to perform the simultaneous detection of the three analytes. 

#### 3.2.3. Investigation of the Influence of the Scan Rate on Kinetics of DA Detection 

**Figure 4:**
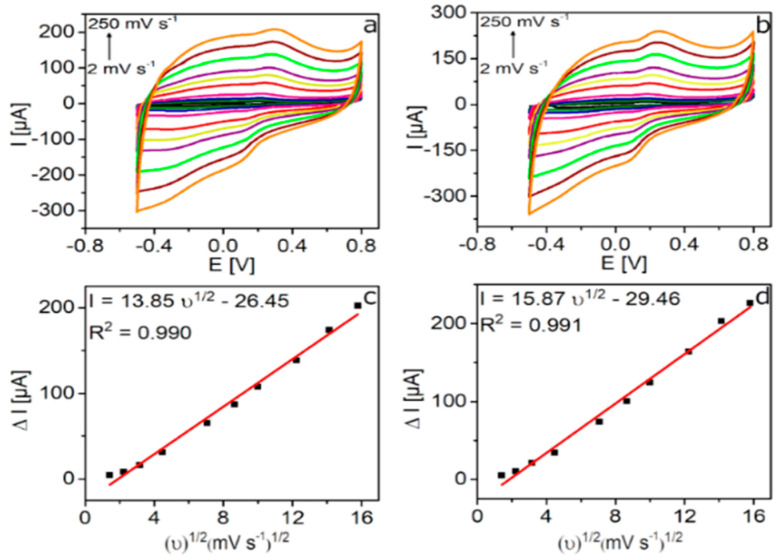
CVs recorded on (**a**) GCE/rGO-Au and (**b**) GCE/N-rGO-Au electrodes at different scan rates (2, 5, 10, 20, 50, 75, 100, 150, 200, 250 Mv·s^−1^) in 0.1 PBS (pH 7.0) with 100 µM DA. Dependence of maximum anodic current against the square root of the scan rate for (**c**) GCE/rGO-Au and (**d**) GCE/N-rGO-Au.

The effect of the potential scan rate on the oxidation current of the GCE/rGO-Au and GCE/N-rGO-Au electrodes in 0.1 PBS (pH 7.0) containing 100 µM of DA was investigated in the scan rate range of 2-250 Mv·s^−1^ (Figure 4). Even though the anodic current and peak potential increased with increasing the scan rate for both electrodes, the better defined DA oxidation peaks (presenting higher current values and lower overpotentials) were developed on the GCE/N-rGO-Au electrode (Figure 4b). In addition, the capacitive current is always lower on this electrode. Figure 4c,d show the relationship between the maximum anodic currents measured on both electrodes and the square root of the scan rates, which are in agreement with the DA oxidation mechanism mainly controlled by diffusion [35]. 

#### 3.2.4. Simultaneous Determination of DA, AA, and UA

**Figure 5:**
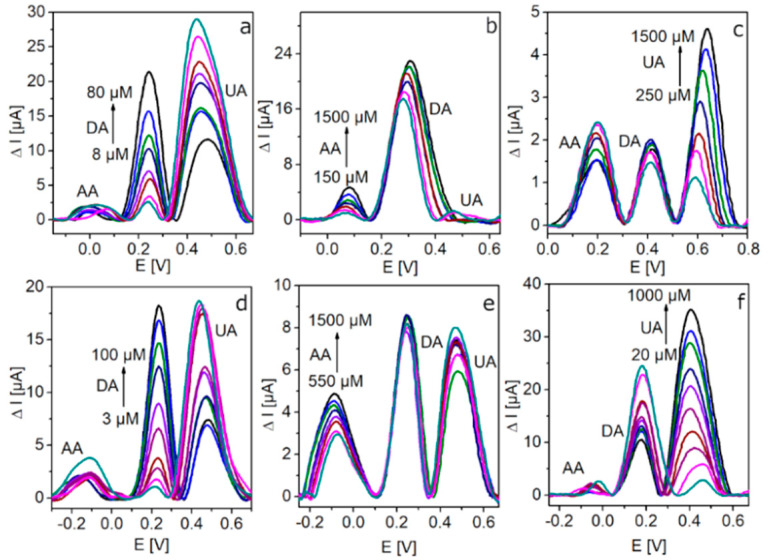
DPVs recorded on the GCE/rGO-Au (**a**–**c**) and GCE/N-rGO-Au (**d**–**f**) electrodes in the presence of DA, AA, and UA in 0.1 PBS (7.0). (**a**,**d**) 300 µM UA, 300 µM AA, and increasing concentrations of DA; (**b**,**e**) 100 µM DA, 300 µM UA, and increasing concentration of AA; (**c**,**f**) 100 µM DA, 300 µM AA, and increasing concentration of UA.

DPV experiments were carried out to investigate the simultaneous detection of DA, AA, and UA, as this electrochemical technique is highly sensitive and responsive towards Faradaic processes [36]. Figure 5 presents the baseline corrected DPVs recorded on GCE/rGO-Au and GCE/N-rGO-Au electrodes in a 0.1 M PBS solution (pH 7.0) containing DA, AA, and UA. The concentration of the target analyte was increased while those of the other two species were kept constant. As it can be seen, the oxidation peaks of the three analytes are well-separated, showing peak potential separation values of 0.34, 0.20, and 0.54 V for AA-DA, DA-UA, and AA-UA on the GCE/N-rGO-Au electrode, respectively indicating a suitable detection of one analyte in the presence of the other two species. Figure S6 shows the linear relationship between the oxidation peak currents and the concentrations of DA, AA, and UA. Very high correlation coefficients (*R*^2^ = 0.989–0.995) prove excellent fitting of the experimental data to the obtained equations. As expected, the anodic peak currents corresponding to the different analytes (mainly DA and UA) increased linearly with the concentrations for GCE/N-rGO-Au and GCE/rGO-Au. Regarding DA detection (Figure 5a,d), when its concentration increases (with a subsequent enhancement of the corresponding anodic current), the UA oxidation peak decreases while the AA remains unaffected, suggesting the suitable quantitative analysis of DA in the presence of UA and AA using both electrodes. However, GCE/N-rGO-Au exhibits a wider linear range determined for the DA sensing. Figure 5b,e shows the Faradaic responses of the two electrodes towards AA increasing concentrations (keeping constant the concentrations corresponding to the other two analytes). Even though on both electrodes, the anodic current corresponding to AA oxidation increases with concentration, the simultaneous detection of the three analytes under study is only possible on the GCE/N-rGO-Au electrode as the signal related to UA detection is negligible on GCE/rGO-Au (Figure 5b). The linear range for GCE/N-rGO-Au is narrower than that recorded on GCE/rGO-Au (550–1500 vs. 100–1500 µM). It can be the result of the intense occupation of the active sites mainly by DA and UA molecules present in the solution. Finally, Figure 5c,f shows the DPVs recorded at increasing UA concentration, keeping constant the concentration of DA and AA. The oxidation peak current corresponding to UA increases on both electrodes while the corresponding to DA anodic process decreases. However, the linear range related to UA detection has a lower bottom value on GCE/N-rGO-Au, also probing the better sensing performance of this electrode. Table 3 summarizes the LODs, linear ranges, and sensitivity values of the electrodes with respect to DA, AA, and UA, according to the results from DPV analysis. The LOD values for both electrodes (signal-to-noise ratio, S/N = 3) were calculated according to the Equation:

LOD = 3S/b(1)

where S is the standard deviation of the blank sample and b is the slope of the calibration curve. GCE/N-rGO-Au has a lower LOD value for DA compared to GCE/rGO-Au (2.4 vs 3.9 µM); similarly, the LOD values for UA are 8.7 vs. 68 µM. This better sensing performance of the GCE/N-rGO-Au electrode towards the simultaneous detection of DA, AA, and UA could be explained by homogeneous distribution of AuNPs on the graphene material surface (Figure 1), confirming the positive contribution of the N atoms. The calibration curves corresponding to the different analytes together with the related linear regression equations (Figure S6) are in agreement with mainly diffusion-controlled mechanisms. According to these results, the linear range for DA detection on GCE/N-rGO-Au was significantly wider (3–100 µM vs. 8–80 µM for GCE/rGO-Au). This finding indicates the beneficial role of nitrogen in the graphene material, which facilitates the high dispersion of AuNPs and increases their electrocatalytic activity [29]. In contrast, GCE/rGO-Au has wider linear ranges for detecting UA and AA compared to GCE/N-rGO-Au (Table 3). Furthermore, DPV measurements were also performed in solutions containing each analyte separately for comparative purposes (Figure S7, and calibration curves Figure S8). The fabricated GCE/N-rGO-Au electrode exhibits a very low detection limit towards DA in the absence of interfering species, making this value almost twofold lower in comparison to GCE/rGO-Au (385 nM vs. 700 nM). In addition, the LODs for AA and UA were also improved on this electrode when comparing to GCE/rGO-Au (44 and 2.7 µM, respectively, for GCE/N-rGO-Au and 56 and 17 µM for GCE/rGO-Au). Although the LOD values are higher in the presence of the interfering species (Table 3), the simultaneous detection of DA, AA, and UA was still possible with satisfactory results. Regarding the sensitivity of the different sensors evaluated, Table 3 reveals that the GCE/rGO-Au-based sensor is more sensitive than the N-doped to DA. This is in agreement with previously published results as the oxygen content of GCE/rGO-Au is higher than that of GCE/N-rGO-Au (14.6 vs. 9.0 at.%), indicating stronger interactions between cationic DA molecules and electronegative oxygen functionalities on the surface of GCE/rGO-Au at pH = 7.0 [25]. Interestingly, the GCE/N-rGO-Au electrode is more sensitive towards UA, which may be explained by the more homogeneous dispersion of AuNPs and/or the presence of nitrogen atoms in the active material of electrode. However, both modified electrodes exhibit very low sensitivity towards AA, which can be explained by residual AuCl^4−^ ions on the surface of the electrode participating in AuNPs formation as AA is commonly used as a reducing agent for HAuCl_4_ during the synthesis of AuNPs [29]. 

#### 3.2.5. Reproducibility, Stability, and Selectivity of the GCE/N-rGO-Au Electrodes

**Figure 6:**
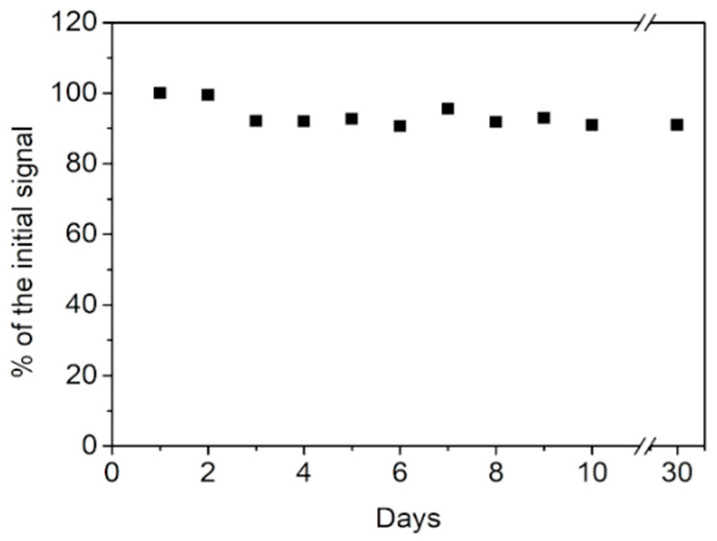
Long-term stability of the GCE/N-rGO-Au sensor in 100 µM DA.

The reproducibility of the GCE/N-rGO-Au electrode towards DA oxidation was evaluated by comparing the anodic responses of five different modified electrodes. The relative standard deviation (RSD) calculated from the data obtained in 0.1 M PBS containing 100 µM DA was 4.3%, thus indicating proper reproducibility. This result demonstrates that AuNPs prevent electrode fouling by oxidized forms of DA [37]. Moreover, the long-term stability of the GCE/N-rGO-Au was evaluated by daily measurements of the DA oxidation peak current for ten days (Figure 6). When the electrode was not being used, it was immersed in 0.1 PBS (pH = 7.0) and stored at 25 °C. After 10 days, the response of GCE/N-rGO-Au decreased to 87 % of the initial signal. In addition, the electrocatalytic activity of the sensor was verified after one month. The peak current of the GCE/N-rGO-Au sensor remained at a comparable level. The outstanding long-term stability of the GCE/N-rGO-Au sensor in the detection of DA can be explained by the high content and uniform distribution of AuNPs on the N-doped graphene surface, which protects against the oxidation of nitrogen functional groups. 

The selectivity studies were also performed on GCE/N-rGO-Au in the presence of 300 µM of glucose and 100 µM of NaCl as interfering species by means of the standard addition technique (Table 4). Certain amounts of DA, AA, and UA were added into the solution (Table 4). The concentrations of DA, AA, and UA measured maintained average values of 96.7, 79.7, and 80.5 %, respectively, with corresponding RSD values of 3.5, 5.2, and 1.6 %, indicating that GCE/N-rGO-Au is a suitable and selective electrode to determine DA, AA, and UA in the presence of these interferences.

## 4. Conclusions

In this work, a GCE modified with N-rGO-Au nanocomposite is proposed as electrochemical sensor to improve the simultaneous detection of DA, AA, and UA. Compared to rGO, the N-doping facilitates the electrodeposition of a high amount of homogeneously distributed AuNPs due to the high affinity of the metallic particles to the nitrogenated areas of the graphene material. Both GCE/rGO-Au and GCE/N-rGO-Au electrodes exhibit suitable electrocatalytic oxidation activity towards the three tested analytes, showing well-resolved anodic peaks in DPVs. Moreover, the fabricated sensors are suitable to their simultaneous detection, even when the concentration of one analyte exceeds the others. However, the electrochemical sensing performance of the GCE/N-rGO-Au sensor is better than that of the GCE/rGO-Au sensor. The modification of GCE with the N-doped rGO containing AuNPs provides not only wide linear ranges towards DA, AA, and UA (3–100 µM, 550–1500 µM and 20–1000 µM, respectively) but also lower LOD values (2.4, 58, and 8.7 µM vs. 3.9, 57, and 68 µm on GCE/rGO-Au). Furthermore, the GCE/N-rGO-Au electrode is highly stable, maintaining the DA oxidation signal nearly unchanged after one month of storage. This study has demonstrated great potential for the use of N-rGO containing AuNPs as electroactive material suitable in the simultaneous electrochemical detection of DA, AA, and UA.

## Figures and Tables

**Table 1:** Surface chemical composition of rGO, N-rGO, and their nanocomposites with AuNPs determined by XPS (at.%).

Sample	C	N	O	Au
rGO	83.3	-	16.7	-
rGO-Au	76.8	-	14.6	6.2
N-rGO	84.8	6.4	8.8	-
N-rGO-Au	80.7	2.7	9.0	7.6

**Table 2:** Type and amount of oxygen and nitrogen functional groups (at.%).

Sample	C1s Peak Deconvolution				N1s Peak Deconvolution				
	Csp^2^	C-O/C-N	C=O	O=C-OH	N6	NC	N5	NQ	NX
rGO	54.1	18.0	5.7	5.1	-	-	-	-	-
rGO-Au	40.0	22.3	8.5	5.9	-	-	-	-	-
N-rGO	50.3	24.8	7.7	2.0	2.6	0.5	1.9	0.9	0.5
N-rGO-Au	48.9	26.3	4.6	0.9	0.7	1.0	0.7	0.3	-

**Table 3:** Electrochemical performance of GCE/rGO-Au and GCE/N-rGO-Au electrodes towards simultaneous detection of DA, AA, and UA.

Electrodes	LOD [µM]			Linear Range [µM]			Sensitivity [µA µM-1]		
	DA	AA	UA	DA	AA	UA	DA	AA	UA
GCE/rGO-Au	3.9	57	68	8–80	100–1500	250–1500	0.41	0.003	0.003
GCE/N-rGO-Au	2.4	58	8.7	3–100	550–1500	20–1000	0.19	0.002	0.034

**Table 4:** Selectivity studies in the presence of glucose and NaCl as interfering species**.**

Analyte	Added	Measured	Detection Performance	RSD
	[µM]	[µM]	[%]	[%]
DA	50	50.7	101.4	3.5
		46.8	93.6	
		47.5	95.0	
AA	600	485.0	80.8	5.2
		505.0	84.2	
		445.0	74.2	
UA	100	82.4	82.4	1.6
		79.7	79.7	
		79.4	79.4	

## Supplementary Materials

The following are available online at https://www.mdpi.com/1424-8220/20/16/4427/s1, Figure S1: CVs recorded on (a) GCE/rGO-Au and (b) GCE/N-rGO-Au electrodes at different number of electrodeposition cycles in 0.1 M PBS (pH 7.4) with 100 µM DA; Figure S2: Deconvolutions of the C1s core-level XPS spectra of (a) N-rGO, (b) N-rGO-Au, (c) rGO and (d) rGO-Au; Figure S3: Deconvolutions of the N1s core-level XPS spectra of N-rGO and N-rGO-Au; Figure S4: CVs recorded on (a) GCE/rGO-Au and (b) GCE/N-rGO-Au electrodes at different pH values in 0.1 M PBS containing 300 µM AA. Effect of the pH on the anodic peak current and potential values related to the oxidation of AA on (c) GCE/rGO-Au and (d) GCE/N-rGO-Au; CVs recorded on (a) GCE/rGO-Au and (b) GCE/N-rGO-Au electrodes at different pH values in 0.1 M PBS containing 300 µM UA. Effect of the pH on the anodic peak current and potential values related to the oxidation of UA on (c) GCE/rGO-Au and (d) GCE/N-rGO-Au; Figure S5: CVs recorded on (a) GCE/rGO-Au and (b) GCE/N-rGO-Au electrodes at different pH values in 0.1 M PBS containing 300 µM UA. Effect of the pH on the anodic peak current and potential values related to the oxidation of UA on (c) GCE/rGO-Au and (d) GCE/N-rGO-Au; Figure S6: Calibration curves for (a,b,c) GCE/rGO-Au and (d,e,f) GCE/N-rGO-Au for (a,d) DA; (b,e) AA; and (c,f) UA in simultaneous detection; Figure S7: DPVs of the individual detections recorded on the (a,b,c) GCE/rGO-Au and (d,e,f) GCE/N-rGO-Au electrodes in 0.1 PBS (7.0) for (a,d) DA; (b,e) AA; and (c,f) UA; Figure S8: Calibration curves for (a,b,c) GCE/rGO-Au and (d,e,f) GCE/N-rGO-Au electrodes in 0.1 PBS (7.0) for (a,d) DA; (b,e) AA; and (c,f) UA.
