# Supplementary material for: N-Doped Reduced Graphene Oxide/Gold Nanoparticles Composite as an Improved Sensing Platform for Simultaneous Detection of Dopamine, Ascorbic Acid, and Uric Acid
Source: Sensors (Basel). 2020 Aug 7;20(16):4427. doi: 10.3390/s20164427 (PMC7472481; doi:10.3390/s20164427)
Supplement: Supplementary file 1 [file sensors-20-04427-s001.pdf]

Supplementary material

Letter

N-doped reduced graphene oxide/gold nanoparticles composite as an improved sensing platform for simultaneous detection of dopamine, ascorbic acid and uric acid

Daria Minta [1], Zoraida Gonzalez [2], Piotr Wiench [1], Stanisław Gryglewicz [3], Grażyna Gryglewicz [1,*]

[1] Department of Process Engineering and Technology of Polymer and Carbon Materials, Faculty of Chemistry, Wrocław University of Science and Technology, Gdańska 7/9, 50-344 Wrocław, Poland; daria.minta@pwr.edu.pl (D.M.); piotr.wiench@pwr.edu.pl (P.W.)

[2] Instituto de Ciencia y Tecnología del Carbono, INCAR-CSIC, Francisco Pintado Fe, 26, 33011 Oviedo, Spain; zoraidag@incar.csic.es (Z.G.)

[3] Department of Engineering and Technology of Chemical Processes, Faculty of Chemistry, Wrocław University of Science and Technology, Wybrzeże Wyspiańskiego 42, 50-344 Wrocław, Poland; stanislaw.gryglewicz@pwr.edu.pl (S.G.)

* Correspondence: grazyna.gryglewicz@pwr.edu.pl; Tel.: +48-71-320-6398; Fax: +48-71-320-6506

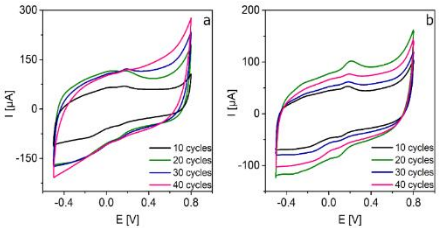

Figure S1. CVs recorded on (a) GCE/rGO-Au and (b) GCE/N-rGO-Au electrodes at different number of electrodeposition cycles in 0.1 M PBS (pH 7.4) with 100 μM DA.

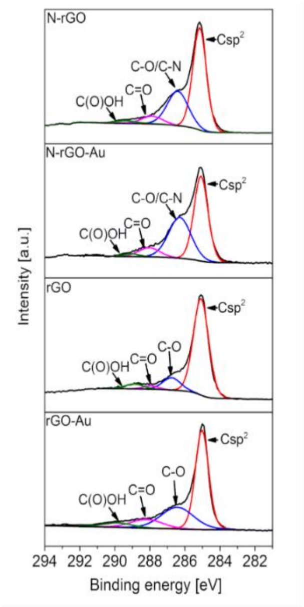

Figure S2. Deconvolutions of the C1s core-level XPS spectra of (a) N-rGO, (b) N-rGO-Au, (c) rGO and (d) rGO-Au.

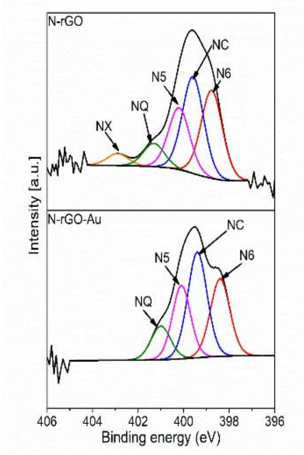

Figure S3. Deconvolutions of the N1s core-level XPS spectra of N-rGO and N-rGO-Au.

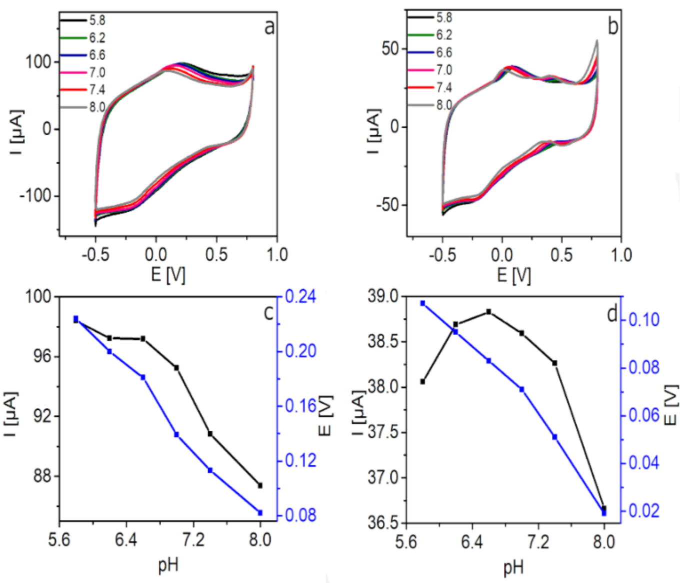

Figure S4. CVs recorded on (a) GCE/rGO-Au and (b) GCE/N-rGO-Au electrodes at different pH values in 0.1 M PBS containing 300 μM AA. Effect of the pH on the anodic peak current and potential values related to the oxidation of AA on (c) GCE/rGO-Au and (d) GCE/N-rGO-Au.

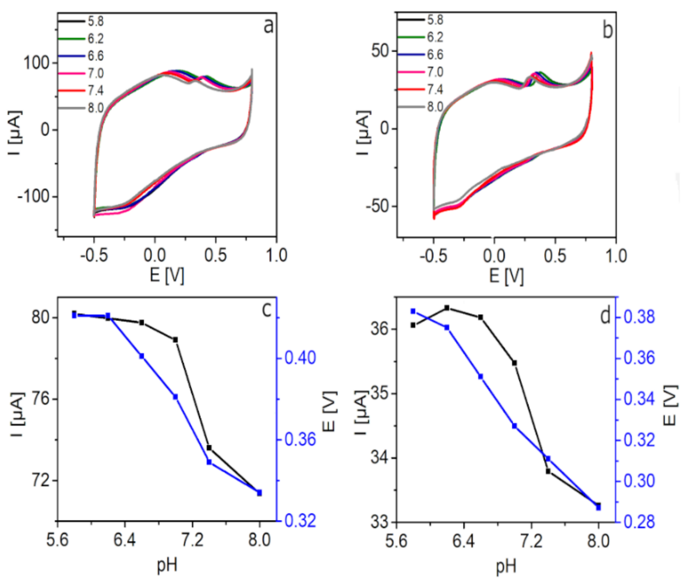

Figure S5. CVs recorded on (a) GCE/rGO-Au and (b) GCE/N-rGO-Au electrodes at different pH values in 0.1 M PBS containing 300 μM UA. Effect of the pH on the anodic peak current and potential values related to the oxidation of UA on (c) GCE/rGO-Au and (d) GCE/N-rGO-Au.

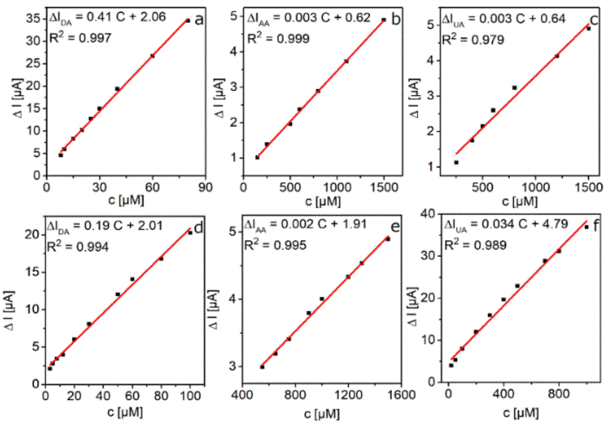

Figure S6. Calibration curves for (a, b, c) GCE/rGO-Au and (d, e, f) GCE/N-rGO-Au for (a, d) DA; (b, e) AA; and (c, f) UA in simultaneous detection.

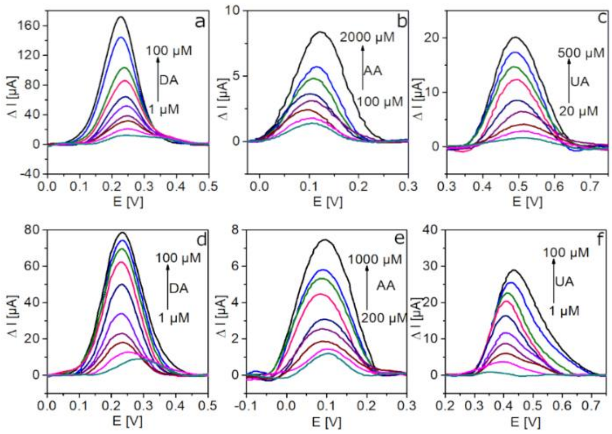

Figure S7. DPVs of the individual detections recorded on the (a, b, c) GCE/rGO-Au and (d, e, f) GCE/N-rGO-Au electrodes in 0.1 PBS (7.0) for (a, d) DA; (b, e) AA; and (c, f) UA.

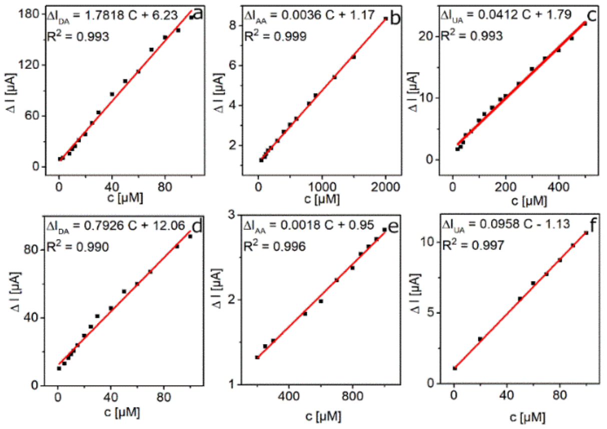

Figure S8. Calibration curves for (a, b, c) GCE/rGO-Au and (d, e, f) GCE/N-rGO-Au electrodes in 0.1 PBS (7.0) for (a, d) DA; (b, e) AA; and (c, f) UA.
